# Efficient Sensitized Photoluminescence from Erbium Chloride Silicate via Interparticle Energy Transfer

**DOI:** 10.3390/ma15031093

**Published:** 2022-01-30

**Authors:** Hao Shen, Huabao Shang, Yuhan Gao, Deren Yang, Dongsheng Li

**Affiliations:** 1 State Key Laboratory of Silicon Materials, School of Materials Science and Engineering, Zhejiang University, Hangzhou 310027, China; 21426020@zju.edu.cn (H.S.); 12026010@zju.edu.cn (H.S.); 11326024@zju.edu.cn (Y.G.); mseyang@zju.edu.cn (D.Y.); 2 Institute of Fluid Physics, China Academy of Engineering Physics, Mianshan Road 64#, Mianyang 621900, China

**Keywords:** interparticle energy transfer, erbium chloride silicate, sensitized emission

## Abstract

In this study, we prepare Erbium compound nanocrystals and Si nanocrystal (Si NC) co-embedded silica film by the sol-gel method. Dual phases of Si and Er chloride silicate (ECS) nanocrystals were coprecipitated within amorphous silica. Effective sensitized emission of Er chloride silicate nanocrystals was realized via interparticle energy transfer between silicon nanocrystal and Er chloride silicate nanocrystals. The influence of density and the distribution of sensitizers and Er compounds on interparticle energy transfer efficiency was discussed. The interparticle energy transfer between the semiconductor and erbium compound nanocrystals offers some important insights into the realization of efficient light emission for silicon-based integrated photonics.

## 1. Introduction

Rare earth ion (RE^3+^)-containing luminescent materials, which possess abundant and sharp emissions via intra-4f transitions, have been gaining considerable interest in many areas such as optical amplifiers for telecommunications [1], solid-state light sources [2], bio-sensors [3], solar cells [4], etc. Among these RE^3+^ ions, erbium ions have been already widely employed in long-haul telecommunications due to their transition from first excited state to ground state emitting a photon at 1.5 μm that coincides with the minimum loss window of silica optical fiber [5]. Since the mid-1980s, silicon-based erbium-doped materials (including Si [6], SiO_x_ [7] SiN_x_ [8], etc.) were considered as a very promising platform for on-chip integrated photonics applications. The optical gain of erbium-containing materials is proportional to the erbium concentration according to a simple two-level model [9]. In order to satisfy the requirement for chip-scale integration application, it is necessary that there should be a much higher erbium doping density compared with Er-doped fiber amplifiers [10]. However, increasing the erbium concentration is limited by low Er solubility in many hosts (for instance, ~10^20^ cm^−3^ in silica [11]). The concentration quenching effect would be severe owing to the formation of optically inactive erbium precipitates and clusters inside the host when the doping density exceeds solid solubility [12]. Crystalline erbium compounds, such as Er_2_Si_2_O_7_ [13] and erbium chloride silicate (ECS) [14], have been expected to overcome the solubility limit because erbium ions are periodically arranged at lattice sites. For example, giant net optical gain over 100 dB/cm has been realized in single-crystal ECS nanowires [15]. 

Although erbium compounds with high erbium content have great potential in chip-scale integration, they also suffer from very low excitation cross section owing to the parity-forbidden intra-4f transitions of Er^3+^ [16]. In general, silicon nanocrystals (Si NCs) that absorb broadband excitation light and then transfer energy to a nearby Er^3+^ can be introduced conveniently inside Er-doped materials as sensitizers [17]. Experimental data that the effective cross section of film embedded with Si NCs receive a nearly five of orders magnitude enhancement, confirming the high efficiency of this energy transfer process [18]. However, the strategy of co-doping Si NC cannot work in crystalline erbium compounds in view of the physical separation of Si NCs and erbium compounds [19,20]. The distance between Si NC and Er^3+^ is lower than the energy transfer distance because the Er^3+^ ions in the crystalline compound are fixed in the lattice position rather than distributed in the amorphous matrix. To overcome the separation, researchers attempted to constrain sensitizers and active centers into a core–shell nanostructure [21,22] or synthesize free-standing nanoparticles followed by incorporating them into a colloidal mixture for interparticle energy transfer (IPET) [23,24]. Although some exciting results have been reported based on energy transfer in core–shell or interparticle nanostructure, there are some limitations that prevent these methods from practical application, such as complex fabrication procedures and quenching effects induced by surface state [25]. Simultaneously tailoring sensitizers and active center phase precipitation in a solid matrix may be a possible solution to the abovementioned problems, but the lattice and dimension mismatch between Si NC and Er compounds restrict the IPET process inside host materials [19]. To the best of our knowledge, there is no report of sensitized crystalline Er compound emissions via IPET inside the matrix. Moreover, it is unknown how the density and distribution of sensitizers and Er compounds in host material affect the IPET efficiency. Here, we fabricate the amorphous silica film with Si NCs and ECS nanocrystals embedded using a sol-gel method. We demonstrate that the efficient sensitized emission of crystalline ECS can be achieved via interparticle energy transfer from Si NCs. Additionally, the influence of density and the distribution of sensitizers and Er compounds on IPET efficiency were discussed based on different Er^3+^ concentrations.

## 2. Materials and Methods 

The Er compound nanocrystal and Si NC co-embedded silica film was prepared by the sol-gel process followed by spin-on method. The Si NC was obtained from thermal dissociation of Si-H bonds generated by the hydrolysis–condensation reactions of triethoxysilane (H-Si(OC_2_H_5_)_3_, TES) in an annealing process. At first, TES was dissolved in ethanol and de-ionized water to form silica sol with excess silicon. Then, different amounts of erbium chloride solution with 0–40% molar ratio of erbium to silicon were added to the silica sol as a precursor of Er compound nanocrystals. The diluted HCl was added dropwise to the mixture solution under vigorous stirring for adjusting the PH to 5. After a complex hydrolysis–condensation reaction, the uniform pink sol containing Er^3+^ and excess silicon was formed. Subsequently, the as-prepared sol was spin-coated on a clean p-Si substrate, followed by a careful drying process for liquid solvent removal at 80 °C. Finally, all the samples were annealed for one hour at 1000 °C in a tube furnace under Ar atmosphere. The reference sample with no excess silicon, which was prepared by substituting precursor TES with tetraethyl orthosilicate (Si(OC_2_H_5_)_4_, TEOS), was treated with the same annealing procedure and labeled as 10% TEOS.

To identify the structures and crystallinity of the Er-related phase, X-ray diffraction (XRD) data were collected on an X-ray diffractometer (Rigaku D/max-2550pc, Rigaku Company, Tokyo, Japan) equipped with a Cu *Kα* radiation source (λ = 0.154 nm). Cross-section samples prepared were observed under an electron microscope to study the microstructure and crystalline size. The specific preparation method is as follows: the samples were bonded with M-bond 610 and double-sided thinned to reduce the thickness to less than 20 microns. The samples were made into a thin area using a Gatan 691 ion-thinning instrument. Then, the cross-section samples were studied by using a TECHNAI-F20G2 transmission electron microscope (TEM, FEI Company, Hillsboro, OR, USA) under 200 keV accelerating voltage. The line resolution of the TEM is 0.102 nm, and the point resolution is 0.24 nm. Steady-state PL in the visible and near-infrared range is detected using a charge-coupled device (PIXIS:100BR, Princeton Instruments company, America) and InGaAs photomultiplier tube (PMT, R5509, Hamamatsu Company, Hamamatsu, Japan), respectively. Two lasers with wavelengths of 473 nm and 980 nm were used as a light source for distinguishing sensitized Er^3+^ emission. Time-resolved visible PL spectra were recorded on an Photonics FLS spectrophotometer (Edinburgh Instruments company, Britain), equipped with a μF 920 adjustable microsecond lamp as the excitation source.

## 3. Results and Discussion

**Figure 1:**
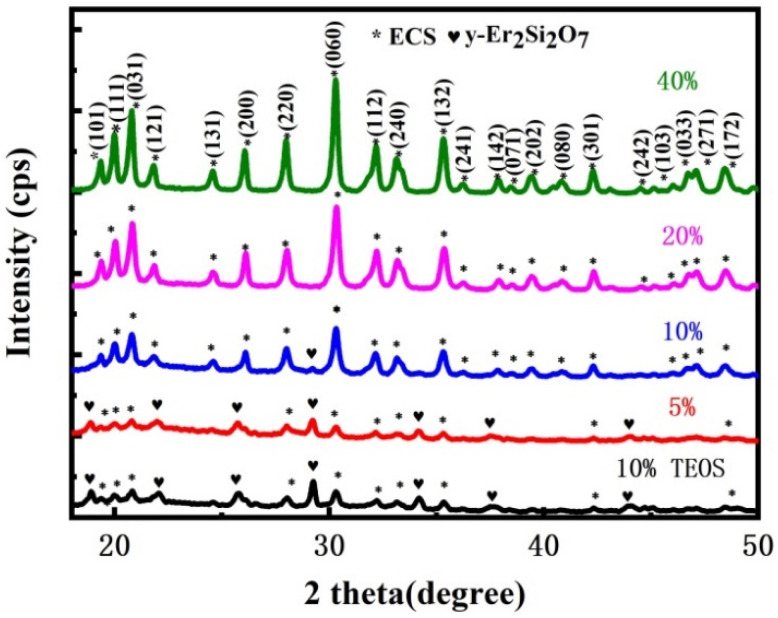
XRD patterns of reference sample with TEOS as precursor and samples with different Er^3+^ concentrations. The asterisks and diamonds represent the diffraction peaks of ECS and y-Er_2_Si_2_O_7_, respectively.

XRD patterns of the reference sample and samples with different Er^3+^ concentrations are provided in Figure 1. XRD analysis illustrated that crystalline structures exist in all samples. It is apparent that the crystallization peaks shall be assigned to the Er-related phase since the diffraction peak intensity increases with Er^3+^ concentration. Here, the observed stronger diffraction peaks, which occurred in higher Er^3+^ concentration samples, indicate increased growth and crystallinity of matrix-embedded Er-related crystals. In other words, the diameter of Er-related crystal shall increase with Er^3+^ concentration, which is consistent with the other system of crystal embedded in amorphous matrix [26]. For samples with a higher Er:Si ratio, the diffraction peaks match well with erbium chloride silicate (ECS) (JCPDS No. 042-0365, Pnma), which exhibited excellent optical properties in the form of nanowire and nanosheet [27,28]. The main peaks of the ECS phase are marked with asterisks and crystal plane indices.

It is noteworthy that the reference sample and samples with a lower Er^3+^ concentration show some new diffraction peaks in addition to ECS crystals, which were marked with diamonds shown in Figure 1. These peaks are supposed to arise from erbium oxide or silicate, but there is no known Er-related phase in the JCPDS reference database fully corresponding to the XRD pattern. Other RE^3+^-related phases, especially those with a close ionic radius to Er^3+^, may be retrieved for reference as the crystal structures of rare earth oxide and silicate are dependent on ionic radius. This method was proved to be appropriate in previous studies [29,30]. According to the fact that the ionic radii of Er^3+^ and Y^3+^ are nearly the same, it is reasonable to attribute these new diffraction peaks to y-Er_2_Si_2_O_7_ in the context of the large similarity between y-Y_2_Si_2_O_7_ (JCPDS No. 74-1994) and observed peaks [29]. The appearance of y-Er_2_Si_2_O_7_ phases in samples with lower Er^3+^ concentration is likely to be related to the local Er:Si molar ratio. When the Er^3+^ concentration is too low, local Er:Si molar ratio is not large enough for the formation of pure ECS with a 3:2 Er:Si ratio, as reported in Er-doped silica film deposited by magnetron sputtering [31]. Therefore, the reference sample and samples with a lower Er^3+^ concentration are composed of a mixture of ECS and y-Er_2_Si_2_O_7_, and the reference sample contains more y-Er_2_Si_2_O_7_ than ECS due to a fairly strong diffraction peak of y-Er_2_Si_2_O_7_. 

**Figure 2:**
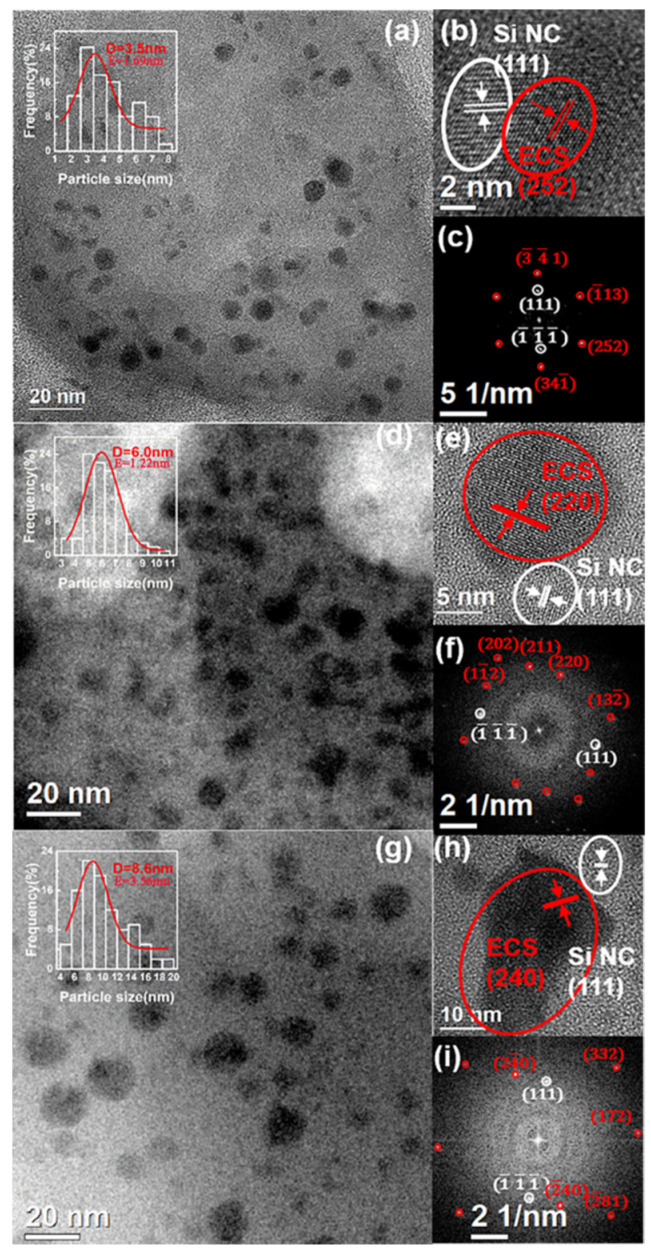
TEM images (inset is the nanoparticle size distribution histogram), HRTEM images of two nearby nanoparticle and the corresponding FFT patterns of the sample with different Er^3+^ concentrations: (**a**–**c**) 10% Er:Si ratio; (**d**–**f**) 20% Er:Si ratio; (**g**–**i**) 40% Er:Si ratio.

In order to further analyze the microstructure and crystallization phase, we performed TEM and HRTEM, including fast Fourier transform (FFT) of HRTEM. As shown in Figure 2a,d,g, TEM images of samples with different Er^3+^ concentrations display that a large number of dark nanoparticles are precipitated within the light matrix, while the distributions of nanoparticles are not very homogenous. It is apparent that the average size of these nanoparticles increases with increasing Er^3+^ concentrations, that is, the average diameter of these nanoparticles increases from 3.6 nm to 8.6 nm with the Er:Si ratio increasing from 10% to 40%. 

The HRTEM images as shown in Figure 2b,e,h demonstrate that there is another type of nanocrystal near to some dark nanoparticles and confirm their crystalline nature with high crystallinity. The measured interplanar spacings of dark nanoparticles are 1.92, 3.18 and 2.70 Å, consistent with the (252), (220) and (240) crystalline planes of orthorhombic structure of ECS, respectively. On the other hand, all the lighter nanocrystals present a lattice spacing of about 3.1 Å that is well matched with the (111) interplane distance of the diamond structure of Si. Although the measured interplane spacings of these nanocrystals are in good agreement with the designated crystalline planes, the confirmation of the phase structure would require a more in-depth analysis owing to the close interplane spacing within different Er-related phases and silicon. FFT patterns of corresponding HRTEM images further demonstrate that these two types of nanoparticles are attributed to be ECS and Si NC, and the determined Miller indices of crystalline planes were marked in Figure 2c,f,i. 

The indirect excitation of Er^3+^ in Er-compound crystalline is difficult to achieve because it is an intriguing challenge to simultaneously control the crystallization of two types of phases in the neighborhood within a solid matrix. For example, it was found that the crystallization of Er silicates annealed at higher temperatures lead to the disappearance of Si NC formed at low annealing temperatures in Er-doped Si-rich silica film [19]. In our system, samples with different erbium concentrations have obtained both Si nanoparticle and ECS nanoparticle crystalline structure, and the distance between the particles is very close. The main reason is that the size difference between Si nanoparticles and ECS nanoparticles in our system is not too large, so the interface of the particles has relatively little effect on the crystallization of the particles. Secondly, the lattice mismatch of ECS relative to erbium silicate and Si is smaller. It seems possible that the Si NCs observed in our system act as sensitizers for Er^3+^ emission, as analogous to those Er-doped materials [32]. 

**Figure 3:**
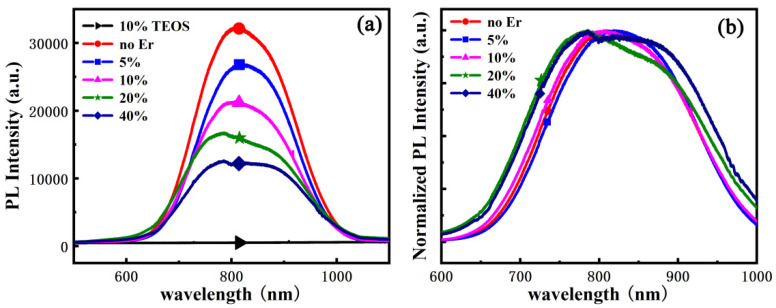
(**a**) The visible range PL spectra of reference sample and samples with different Er:Si ratio (0–40%), (**b**) normalized PL spectra of (**a**) excluding reference sample.

In order to identify the spectral overlap between sensitizers and ECS as well as the concentration of sensitizers, steady-state and time-resolved PL in visible range were carried out. Figure 3a shows the PL spectra of reference sample and samples with a different Er:Si ratio. In the spectra from samples with a different Er:Si ratio, the broad PL spectra peaks at about 800 nm are observed. This emission band may be attributed to confined exciton recombination from Si NC, which is in line with previous studies [16,33]. On the other hand, no PL signal is found from the reference sample due to no excess Si in the film. At first glance, there is no obvious shift of PL peak as the Er:Si ratio increases. The normalized PL spectra shown in Figure 3b further revealed that the emission peaks are almost independent of Er^3+^ concentration, which can be explained as the nearly constant average sizes of Si NCs, whereas the spectral bandwidths of Si NC emissions slightly increase with increasing the Er:Si ratio. A possible explanation for this might be that the Si NC size distribution in samples with a higher Er:Si ratio is broader than those with a lower Er:Si ratio owing to the interaction between Si NC and Er^3+^ ions, since the luminescence energy as well as the PL peak are determined by the size of Si NC [16]. 

In addition to emission peak and spectral bandwidth, it is also apparent from Figure 3a that the signal from Si NC at about 800 nm decreases with increasing the Er^3+^ concentration. This result may partly be explained by the reduction in Si NC concentration within amorphous silica. As the Er^3+^ concentration increases, more and more ECS were generated by reaction with silicon and oxygen during thermal treatment, leading to a slight reduction in excess silicon for Si NC precipitation. More importantly, the decrease in luminescence from Si NC may be the result of the fact that a growing number of excitons confined in the Si NCs transfer energy to ECS and no longer participate in the visible luminescence due to the increased amount of ECS with increasing the Er^3+^ concentration. If the energy transfer from Si NC to ECS occurs as expected, the total de-excitation rate of Si NC will increase after Er^3+^ doping because another non-radiative recombination pathway was introduced. It can thus be suggested that not only the emissions of Si NCs decrease, but also its decay time as the Er^3+^ concentration increases.

**Figure 4:**
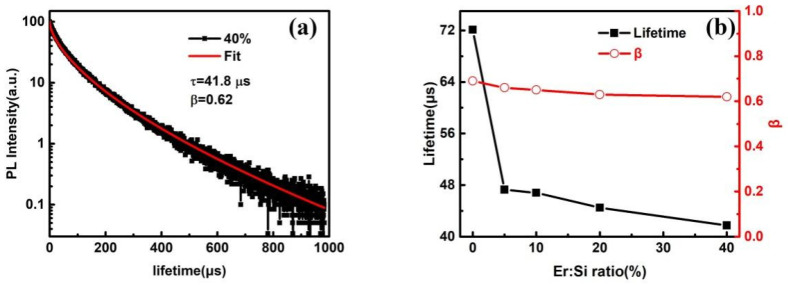
(**a**) PL decay curve of Si NC-related luminescence at 800 nm of sample with 10% Er:Si ratio and corresponding fit curve by stretched exponential function, (**b**) lifetimes and distribution factors (*β*) extracted from stretched exponential function of samples with different Er:Si ratio.

In order to confirm the aforementioned energy transfer process, time-resolved PL measurements of the Si NC emission were performed, and a typical decay curve was displayed in Figure 4a. The PL decay curve is not a single exponential and can be well described by a stretched exponential function like those Si NC embedded in silica [34]:

(1) $$I(t)=I_{0}\exp[-{\left(t/\tau \right)}^{\beta }]$$

where *I*(*t*) and *I*_0_ are the PL intensity as a function of time and at *t* = 0, *τ* represents the mean luminescence lifetime, and *β* (0 < *β* ≤ 1) is a dispersion factor that describes the distribution of lifetimes which becomes broader as *β* decreases to 0. Figure 4b provides the lifetimes and distribution factors fitted with stretched exponential as a function of Er:Si ratio. The lifetime decreases from 72.1 to 41.8 μs and the dispersion factor also decreases from 0.69 to 0.62 as the Er:Si ratio increases from 0 to 40%. The values of tens of μs are in accordance with the lifetimes observed from Si NC [34]. Again, the trend observed in the lifetime identifies an additional recombination channel that most likely is the energy transfer from Si NC to ECS, as reported in other sensitization Er^3+^ emission system [35,36]. The energy transfer efficiency (ETE) is the fraction of donors that are depopulated by energy transfer process to acceptors over the total number of donors being excited, which can be expressed as a function of the PL decay lifetimes, shown below:

(2) $$\eta_{Er}=1-\frac{\tau_{Er}}{\tau_{Er\text{-}free}}$$

The ETE is calculated as 34.4%, 35.1%, 38.3%, and 42.0% for Er:Si ratios of 5%, 10%, 20%, and 40% samples, respectively. Note that the slight decrease in dispersion factor with increasing Er:Si ratio is clear-cut evidence of inhomogeneous distribution of the lifetime caused by broad Si NC size distribution.

**Figure 5:**
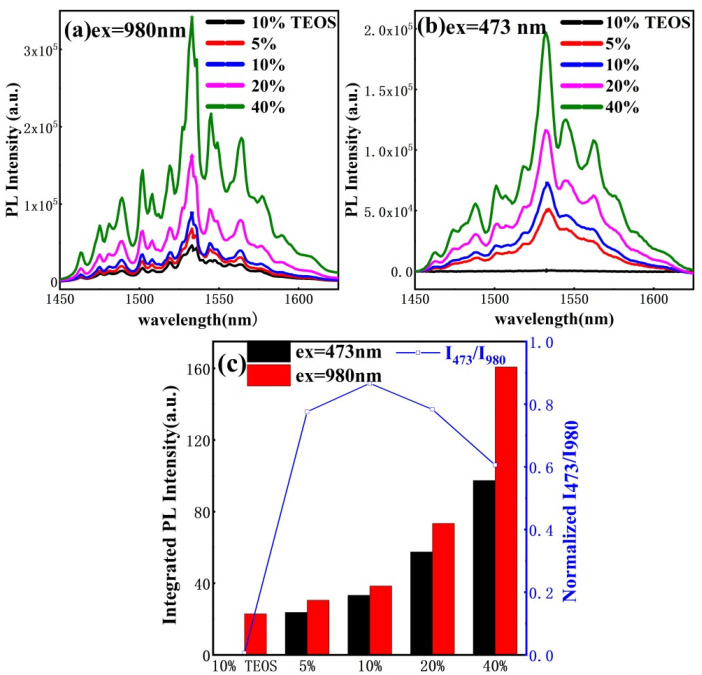
Near-infrared range PL spectra of reference sample and samples with different Er:Si ratio excited by (**a**) 980 nm laser and (**b**) 473 nm laser; (**c**) Integrated PL intensity and the ratio of integrated intensity excited by 473 nm to that by 980 nm laser (I_473_/I_980_).

The occurrence of an energy-transfer process from Si NC to ECS can be investigated by using both resonate and non-resonate wavelength lasers as excitation sources. Figure 5a presents the PL spectra of the reference sample and samples with different Er:Si ratios under resonant wavelength (980 nm) excitation. Several sharp peaks with the main peak at 1533 nm that share almost the same spectrum shape are observed in all samples, similar to the spectrum of single-crystal ECS nanowire in a previous study [14]. These peaks arise from the transition between the Stark levels of ground and first state, indicating that the optically active Er^3+^ ions are mainly in a crystalline environment. For the reference sample and samples with a 5% Er:Si ratio, some weak peaks at 1536, 1540 and 1556 nm ascribed to the emission of y-Er_2_Si_2_O_7_ can be seen [31], which is consistent with the XRD results. It is clear that the PL intensity of the samples increases with the increasing Er:Si ratio, which could be attributed to the increased optically active Er^3+^ concentration. It is noticed that the PL intensity of reference sample is even weaker than samples with 5% Er:Si ratio. This result demonstrates to some extent that no extra non-radiative recombination pathway will form after the introduction of Si NC via precursor TES. On the other hand, it is also suggested that ECS yields much stronger luminescence than y-Er_2_Si_2_O_7_ because the PL spectrum of the reference sample agrees well with PL peaks from ECS in the context of a much larger proportion of y-Er_2_Si_2_O_7_ than ECS phase [31].

Figure 5b shows the PL spectra of the reference sample and samples with different Er:Si ratios obtained using a 473 nm line laser. It is apparent that no PL signal was observed from the reference sample, while all samples with a different Er:Si ratio exhibited intense emission peaks characteristic of Er^3+^ ions. Note that the excitation wavelength of 473 nm does not coincide with any absorption band of Er^3+^, and hence the Er^3+^ can only be excited through a carrier-mediated process. Accordingly, the Er^3+^ in the reference sample cannot be excited under 473 nm pumping owing to the lack of photo-injected carriers, as shown in Figure 3a. In contrast to the reference sample, samples with different Er:Si ratios all contain Si NCs that are able to act as sensitizers for Er^3+^. In addition, there is a competition between Er^3+^ and Si NC luminescence, that is, Er^3+^ emission increases with increasing Er:Si ratio accompanying a quenching of Si NC emission discussed above. These results provide strong evidence of an effective energy transfer from the excitons confined in Si NC to the Er^3+^. Energy transfer from silicon nanocrystals to ECS nanocrystals belongs to that from semiconductor nanocrystals to dielectric nanocrystals, the emission spectrum of the donor is band-shaped and the absorption spectrum of the acceptor is line-shaped, meeting spectral overlap requirements. Under 473 nm optical pumping, electrons and holes confined in silicon nanocrystals are formed, and the peak of released energy by exciton recombination (~1.55 eV in our system) overlaps with the resonance absorption peak of erbium ions (1.53 eV). In this way, erbium ions are excited from the ground excitation state to the third excitation state (^4^*I*_9/2_), and finally show 1.55 μm luminescence after multiple transition processes [37]. It is important to note that the spectrum shape observed under 473 nm pumping is identical to that obtained with the 980 nm pump beam, indicating that the environment of excited Er^3+^ with resonant or non-resonant wavelength pumping is the same. In other words, the sensitized emission of crystalline ECS was acquired based on the interparticle energy transfer between Si NC and ECS. 

As shown in Figure 5c, integrated PL intensitis excited by 473 and 980 nm all increase with increasing Er:Si ratio monotonically, whereas the ratio of them (I_473_/I_980_) displays a slight increase followed by a decline. Integrated PL intensity under 473 and 980 nm pumping are proportional to the Er^3+^ density excited by IPET from Si NC and the total optically active Er^3+^ density, respectively. Therefore, the term I_473_/I_980_ stands for the relative proportion of sensitized Er^3+^ ions and can be used for comparing the sensitized emission between different samples. The I_473_/I_980_ of the sample with a 10% Er:Si ratio is highest among these samples because it owns an optimal balance, that is, IPET from Si NC to ECS in samples with lower Er:Si ratio may suffer from low encounter probability of Si NC and ECS caused by low ECS concentration, while for samples with a higher Er:Si ratio, a possible explanation is that the relative interparticle distance between Si NC and ECS increases due to the increased size of ECS. Effective sensitized emission of Er chloride silicate nanocrystals was realized through interparticle energy transfer between silicon nanocrystals and Er chloride silicate nanocrystals co-embedded in amorphous silicon oxide. In addition, the influence of density and distribution of sensitizers and the Er compound on interparticle energy transfer efficiency was studied. This provides some insights for the sensitization of erbium ions through the particle energy transfer co-embedded in the matrix.

## 4. Conclusions

In conclusion, silicon-rich silicon oxide films doped with a high Er^3+^ concentration have been prepared using the sol-gel method with TES as precursor. Dual phases of Si and Er chloride silicate (ECS) nanocrystals were coprecipitated within amorphous silicon oxide during thermal treatment, and ECS was localized at the vicinity of Si NC. The efficient sensitized luminescence of crystalline (ECS) was achieved via an interparticle energy transfer (IPET) from Si NC to ECS. PL spectra under different excitations have confirmed that samples with a medium Er^3+^ concentration, e.g., 10% Er:Si ratio, holds the optimal sensitization that benefited from the efficient IPET process mainly as a result of the fine distribution of dual phase nanocrystals.

## Data Availability

All experimental data to support the findings of this study are available upon request by contacting the corresponding authors.
